# Rifaximin Improves *Clostridium difficile* Toxin A-Induced Toxicity in Caco-2 Cells by the PXR-Dependent TLR4/MyD88/NF-κB Pathway

**DOI:** 10.3389/fphar.2016.00120

**Published:** 2016-05-09

**Authors:** Giuseppe Esposito, Nicola Nobile, Stefano Gigli, Luisa Seguella, Marcella Pesce, Alessandra d’Alessandro, Eugenia Bruzzese, Elena Capoccia, Luca Steardo, Rosario Cuomo, Giovanni Sarnelli

**Affiliations:** ^1^Department of Physiology and Pharmacology “Vittorio Erspamer”, Sapienza University of RomeRome, Italy; ^2^Department of Clinical Medicine and Surgery, University of Naples Federico IINaples, Italy; ^3^Department of Translational Medical Science, University of Naples Federico IINaples, Italy

**Keywords:** Caco-2 cells, *Clostridium difficile* toxin A, pregnane X receptor, rifaximin, pseudomembranous colitis

## Abstract

**Background:** *Clostridium difficile* infections (CDIs) caused by *Clostridium difficile* toxin A (TcdA) lead to severe ulceration, inflammation and bleeding of the colon, and are difficult to treat.

**Aim:** The study aimed to evaluate the effect of rifaximin on TcdA-induced apoptosis in intestinal epithelial cells and investigate the role of PXR in its mechanism of action.

**Methods:** Caco-2 cells were incubated with TcdA and treated with rifaximin (0.1-10 μM) with or without ketoconazole (10 μM). The transepithelial electrical resistance (TEER) and viability of the treated cells was determined. Also, the expression of zona occludens-1 (ZO-1), toll-like receptor 4 (TLR4), Bcl-2-associated X protein (Bax), transforming growth factor-β-activated kinase-1 (TAK1), myeloid differentiation factor 88 (MyD88), and nuclear factor-kappaB (NF-κB) was determined.

**Results:** Rifaximin treatment (0.1, 1.0, and 10 μM) caused a significant and concentration-dependent increase in the TEER of Caco-2 cells (360, 480, and 680% vs. TcdA treatment) 24 h after the treatment and improved their viability (61, 79, and 105%). Treatment also concentration-dependently decreased the expression of Bax protein (-29, -65, and -77%) and increased the expression of ZO-1 (25, 54, and 87%) and occludin (71, 114, and 262%) versus TcdA treatment. The expression of TLR4 (-33, -50, and -75%), MyD88 (-29, -60, and -81%) and TAK1 (-37, -63, and -79%) were also reduced with rifaximin versus TcdA treatment. Ketoconazole treatment inhibited these effects.

**Conclusion:** Rifaximin improved TcdA-induced toxicity in Caco-2 cells by the PXR-dependent TLR4/MyD88/NF-κB pathway mechanism, and may be useful in the treatment of CDIs.

## Introduction

Pseudomembranous colitis is a condition of the large intestine characterized by inflammation and bleeding (Surawicz and McFarland, 1999). It is mainly caused by the anaerobic Gram-positive bacteria, *Clostridium difficile.* These spore producing bacteria colonize the large intestine and produce toxins [*Clostridium difficile* toxin A (TcdA) and *Clostridium difficile* toxin B (TcdB)] which lead to severe diarrhea, colitis, shock and death in severe cases (Rupnik et al., 2009; Leﬄer and Lamont, 2015). The cost of treatment and duration of hospitalization is also significantly increased in affected individuals (Jodlowski et al., 2006). *Clostridium difficile* infections (CDIs) are common in hospital settings due to excessive use of antibiotics, which wash out the normal gastrointestinal flora, making individuals more vulnerable to bacterial attack (Rupnik et al., 2009). Currently available treatment strategies for CDIs include the use of specific antibiotics against *Clostridium difficile*, fecal transplant and surgery (Waltz and Zuckerbraun, 2016). However, treatment of severe and recurrent CDIs remains a challenge, with limited treatment options available (Ebigbo and Messmann, 2013).

Rifaximin, a synthetic analog of rifamycin, is a broad spectrum antibiotic effective against several Gram-positive as well as Gram-negative aerobic and anaerobic bacteria (Scarpignato and Pelosini, 2006). It is poorly absorbed on oral administration and has no systemic adverse events (Scarpignato and Pelosini, 2005). Rifaximin is mainly used for the treatment of travelers’ diarrhea, hepatic encephalopathy, and irritable bowel syndrome (Layer and Andresen, 2010; Sanchez-Delgado and Miquel, 2015; Cash et al., 2016). Besides its antibiotic effect, rifaximin is a gut-specific activator of human pregnane X receptor (PXR), which is a nuclear receptor expressed in the small intestine that is involved in maintaining the integrity of the intestinal epithelial barrier (Ma et al., 2007; Cheng et al., 2010; Wan et al., 2015)

The aim of the present study was to evaluate the effect of rifaximin on TcdA-induced apoptosis, using the Caco-2 cell line as a model for the human intestinal barrier, and to investigate the role of PXR in its mechanism of action.

## Materials and Methods

Caco-2 cells were purchased from European Collection of Cell Cultures (ECACC, Public Health England Porton Down, Salisbury, UK). Cell medium, chemicals and reagents used for cell culture, and TcdA were purchased from Sigma–Aldrich (St. Louis, MO, USA), unless otherwise stated. Instruments, reagents, and materials used for western blot analysis were obtained from Bio-Rad Laboratories (Milan, Italy). Rabbit anti-zona occludens-1 (ZO-1), anti-occludin and anti-glyceraldehyde-3-phosphate dehydrogenase (GAPDH) antibodies were procured from Cell Signaling Technology (Danvers, MA, USA). Rabbit anti-toll-like receptor 4 (TLR4), mouse anti-ZO-1, anti-Bcl-2-associated X protein (Bax), mouse anti-MyD88, rabbit anti-transforming growth factor-β-activated kinase-1 (pTAK1), and mouse anti-TAK1 antibody were purchased from Santa Cruz Biotechnology (Santa Cruz, CA, USA) and horseradish peroxidase (HRP) was obtained from Dako (Milan, Italy). Fluorescein isothiocyanate-conjugated anti-rabbit antibody and Texas red conjugated anti-mouse antibody were purchased from Abcam (Cambridge, UK), and custom oligonucleotides for electrophoretic mobility shift assay (EMSA) analysis were synthesized by TIB Molbiol (Berlin, Germany).

### Cell Culture

Caco-2 cells were cultured in 6-well plates in Dulbecco’s Modified Eagle Medium (DMEM) containing 10% fetal bovine serum (FBS), 1% penicillin–streptomycin, 2 mM L-glutamate, and 1% non-essential amino acids. A total of 1 × 10^6^ cells/well were plated and incubated for 24 h. Upon reaching confluence, the cells were washed three times with phosphate-buffered saline (PBS), detached with trypsin/ethylene diamine tetraacetic acid (EDTA), plated in a 10 cm diameter petri dish and allowed to adhere for further 24 h.

The Caco-2 cells were randomly divided into six groups: vehicle group, 30 ng/ml TcdA group, 30 ng/ml TcdA plus 0.1 μM rifaximin, 30 ng/ml TcdA plus 1 μM rifaximin (Alfa Wasserman S.p.A, Bologna, Italy), 30 ng/ml TcdA plus 10 μM rifaximin, and 30 ng/ml TcdA plus 10 μM rifaximin plus 10 μM PXR antagonist ketoconazole. Rifaximin concentrations were chosen on the basis of previous studies (Terc et al., 2014). Depending upon the experiments, Caco-2 cells were cultured in either 6-well plates or 96-well plates. The cells were treated with different concentrations of rifaximin (0.1–10 μM) and incubated at 37°C for 24 h, followed by TcdA exposure (30 ng/ml) for 24 h.

### Transepithelial Electrical Resistance Measurement

The transepithelial electrical resistance (TEER) of the epithelial cell monolayer was determined using the EVOM volt-ohm meter (World Precision Instruments Germany, Berlin, Germany) according to the method described by Wells et al. (1998). Briefly, cells plated between 14 and 21 days were used for experimentation, and each epithelial cell layer with a TEER value greater than 1000 Ω/cm^2^, was considered to have tight adhesion. TEER was calculated using the following formula: TEER (Ω/cm^2^) = (Total resistance - blank resistance) (Ω) × Area (cm^2^).

### Western Blot Analysis

Protein expression in the Caco-2 cells was evaluated using western blot analysis. Following the treatments, the cells (1 × 10^6^ cells/well) were harvested, washed twice with ice-cold PBS and centrifuged at 180 × *g* for 10 min at 4°C. The pellet of cells obtained after centrifugation was resuspended in 100 μl ice-cold hypotonic lysis buffer [10 mM 4-(2-hydroxyethyl)-1-piperazineethanesulfonic acid (HEPES), 1.5 mM MgCl_2_, 10 mM KCl, 0.5 mM phenylmethylsulphonylfluoride, 1.5 μg/ml soybean trypsin inhibitor, 7 μg/ml pepstatin A, 5 μg/ml leupeptin, 0.1 mM benzamidine and 0.5 mM dithiothreitol (DTT)]. The suspension was rapidly passed through a syringe needle five to six times to lyse the cells and then centrifuged for 15 min at 13,000 × *g* to obtain the cytoplasmic fraction. The proteins from the cytoplasmic fraction were mixed with a non-reducing gel loading buffer [50 mM Tris(hydroxymethyl)aminomethane (Tris), 10% sodium dodecyl sulfate (SDS), 10% glycerol, 2 mg bromophenol/ml] at a 1:1 ratio, and boiled for 3 min followed by centrifugation at 10,000 × *g* for 10 min. The protein concentration was determined using the Bradford assay and 50 μg of each homogenate was used for electrophoresis using polyacrylamide mini gels.

Proteins were transferred to nitrocellulose membranes that were saturated by incubation with 10% non-fat dry milk in 1X PBS overnight at 4°C and then incubated with rabbit anti-ZO-1, rabbit anti-occludin, rabbit anti-TLR4, rabbit anti-Bax, rabbit anti-p-TAK1, mouse anti-TAK1, mouse anti-MyD88, or rabbit anti-GAPDH antibodies, according to standard experimental protocols. Membranes were then incubated with the specific secondary antibodies conjugated to HRP. Immune complexes were identified by enhanced chemiluminescence detection reagents (Amersham Biosciences, Milan, Italy) and the blots were analyzed by scanning densitometry (GS-700 Imaging Densitometer; Bio-Rad, Segrate, Italy). Results were expressed as optical density (OD; arbitrary units; mm^2^) and normalized against the expression of the housekeeping protein GAPDH.

### Immunofluorescence Staining Analysis

Caco-2 cells were harvested, washed with PBS, fixed in 4% formaldehyde in PBS for 15 min and permeabilized with 0.3% Triton-X100 in PBS for 1 h. Two percent bovine serum albumin (BSA) was used to block the non-specific binding sites. The cells were then incubated overnight with mouse anti-ZO-1 (1:100) and rabbit anti-occludin antibody (1:100), or rabbit monoclonal anti-active caspase-3 (1:100; Abcam, Cambridge, UK) and further incubated in the dark with the appropriate secondary antibody (fluorescein isothiocyanate conjugated anti-rabbit or Texas red conjugated anti-mouse). The cells were analyzed using a microscope (Nikon Eclipse 80i), and images were captured by a high-resolution digital camera (Nikon Digital Sight DS-U1). Appropriate negative controls were done by omitting primary or secondary antibodies.

### Cytotoxicity Assay

The 3-[4,5-dimethylthiazol-2-yl]-2,5 diphenyltetrazolium bromide (MTT) assay was used to determine cell proliferation and survival in the Caco-2 cells (Mosmann, 1983). The cells (5 × 10^4^ cells/well) were plated in 96-well plates and allowed to adhere for 3 h. DMEM was then replaced with fresh medium and the cells were untreated or treated with 30 ng/ml TcdA alone or together with increasing concentrations of rifaximin (0.1, 1.0, and 10 μM) dissolved in ultrapure and pyrogen-free sterile vehicle, in the presence or absence of 10 μM ketoconazole. After 4 h, 25 μl MTT (5 mg/ml MTT in DMEM) was added to the cells and the mixture was incubated for a further 3 h at 37°C. Subsequently, the cells were lysed and the dark blue crystals were solubilized using a 100 μl solution containing 50% *N,N*-dimethylformamide and 20% (w/v) SDS (pH 4.5). The OD of each well was determined using a microplate spectrophotometer equipped with a 620 nm filter (PerkinElmer, Inc; Waltham, MA, USA).

### Electrophoretic Mobility Shift Assay

Electrophoretic mobility shift assay was performed to detect nuclear factor-kappaB (NF-κB) activation in Caco-2 cells after TcdA with or without rifaximin treatment. Briefly, 10 mg of cell extracts were incubated in a binding buffer (8 mM HEPES, pH 7.0, 10% glycerol, 20 mM KCl, 4 mM MgCl_2_, 1 mM sodium pyrophosphate) containing 1.0 mg of poly(dI–dC) and γ-^32P^ end-labeled probe. The probe had a sequence as follows: A) 5′AAC TCC GGG AAT TTC CCT GGC CC3′ and B) 5′GGG CCA GGG AAA TTC CCG GAG TT3′. Nuclear extracts were incubated for 15 min with radiolabelled oligonucleotides (2.5–5.0 × 10^4^ cpm) in a 20 ml reaction buffer containing 2 mg poly(dI-dC), 10 mM Tris–HCl (pH 7.5), 100 mM NaCl, 1 mM EDTA, 1 mM DTT 1 mg/ml BSA, and 10% (v/v) glycerol. Nuclear protein-oligonucleotide complexes were resolved by electrophoresis on a 6% non-denaturing polyacrylamide gel in Tris-Borate-EDTA buffer at 150 V for 2 h at 4°C. The gel was dried and autoradiographed with an intensifying screen at -80°C for 20 h. The relative bands were quantified by densitometric scanning with Versadoc (Bio-Rad Laboratories) and a computer program (Quantity One Software, Bio-Rad Laboratories).

#### DNA Fragmentation Assay

Following treatments Caco-2 cells were harvested, lysed with 400 μl sodium chloride EDTA buffer (75 mM NaCl and 25 mM EDTA) containing 1% (w/v) SDS and 2 U/ml proteinase K, and incubated for 2 h at 55°C. Proteins were precipitated by adding 140 μl 5 M NaCl. After centrifugation, DNA in the supernatant was precipitated by addition ethanol and centrifugation was performed again (15 min; 11,000 × *g*). After washing with 70% ethanol (v/v), the DNA was re-suspended in H2O, separated by agarose gel electrophoresis and stained with ethidium bromide.

### Statistical Analysis

Results were expressed as mean ± SEM of *n* = 5 experiments in triplicate. Statistical analysis was performed using parametric one way analysis of variance (ANOVA) and Bonferroni’s *post hoc* test was used for multiple comparisons. *P*-values < 0.05 were considered significant.

## Results

### Transepithelial Electrical Resistance

**FIGURE 1:**
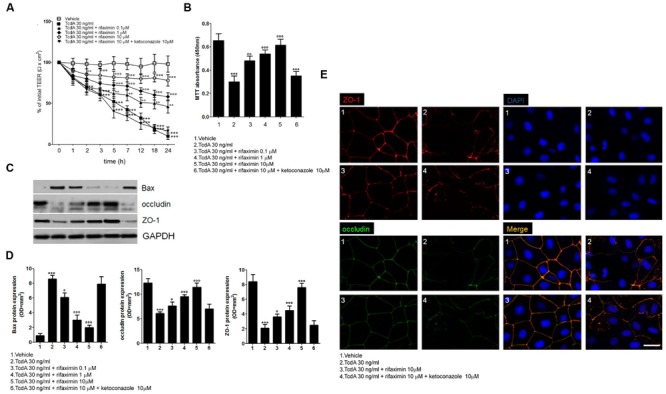
**Effects of increasing concentrations of rifaximin (0.1, 1.0, and 10 μM) alone and rifaximin plus ketoconazole (10 μM) against TcdA (30 ng/ml) in Caco-2 cells: **(A)** 24-h time course TEER changes (*n* = 4); **(B)** MTT cell viability absorbance at 24 h (*n* = 5); **(C)** Immunoreactive bands corresponding to Bax, ZO-1, and occludin expression at 24 h following the TcdA challenge; **(D)** Relative densitometric analysis of immunoreactive bands (arbitrary units normalized against the expression of the housekeeping GAPDH protein; *n* = 5), and **(E)** Immunofluorescent staining showing the effects of TcdA challenge on ZO-1 and occludin co-expression at 24 h.** Nuclei were also investigated using DAPI staining (Scale bar = 25 μm). Results are expressed as mean ± SEM of experiments performed in triplicate. ^∗∗∗^*p* < 0.001 and ^∗∗^*p* < 0.01 vs. vehicle group; ^∘∘∘^*p* < 0.001, ^∘∘^*p* < 0.01 and °*p* < 0.05 vs. TcdA group.

The TEER values in the presence of rifaximin (0.1–10 μM) alone or in the presence of ketoconazole (10 μM) were determined in order to evaluate the barrier integrity of Caco-2 cells exposed to 24 h of TcdA challenge. As seen in **Figure 1A**, a significant time-dependent reduction in TEER was observed starting from 2 h after 30 ng/ml TcdA exposure when compared with the vehicle group. The TEER values at 2, 3, 5, 7, 12, 18, and 24 h were -30, -37, -49, -57, -70, -82, and -91% versus the vehicle group, respectively. Starting at 5 h following the start of the toxin challenge, the effect of TcdA on TEER decrease was significantly counteracted by rifaximin treatment in a concentration-dependent manner. The TEER observed in the 0.1 μM rifaximin group at 5, 7, 12, 18, and 24 h was 19, 43, 56, 177, and 360%, and in the 1.0 μM rifaximin group was 36, 69, 103, 233, and 480%, when compared with the TcdA group. When rifaximin 10 mM was used, TEER reduction was seen starting at 2 h following TcdA stimulus and continued for all the time point intervals (24, 28, 57, 93, 150, 350, and 680% vs. the TcdA group at 2, 3, 5, 7, 12, 18, and 24 h; **Figure 1A**). The effect of rifaximin on the TEER was abolished by the treatment with ketoconazole (**Figure 1A**).

### Cell Viability and Cytotoxicity

As seen in **Figure 1B**, a significant decrease in Caco-2 cell viability (-54%) was observed at 24 h following the TcdA challenge, when compared with the vehicle group (assumed to be 100% viable cells). Under the same experimental conditions, rifaximin caused a significant and concentration-dependent inhibition of cytotoxicity induced by TcdA, resulting in an increased viability of the cultured cells (61, 79, and 105% with 0.1, 1.0, and 10 μM rifaximin, respectively, vs. TcdA group). The effect of rifaximin was almost totally inhibited by ketoconazole (**Figure 1B**).

### Western Blot and Immunofluorescence Staining

The TcdA challenge caused a significant increase in pro-apoptotic Bax protein expression in Caco-2 cell homogenates (955%; *p* < 0.001 vs. vehicle), as seen in **Figures 1C,D**. Treatment with rifaximin resulted in a concentration-dependent decrease in Bax protein expression under the same experimental conditions (-29, -65, and -77% with 0.1, 1.0, and 10 μM rifaximin, respectively, vs. TcdA group). Here again, this anti-apoptotic effect of rifaximin was reverted by ketoconazole (**Figures 1C,D**). To further confirm the ability of rifaximin to significantly affect the TcdA-induced apoptosis also the DNA fragmentation and the expression of caspase-3 were significantly and, in a similar PXR-manner, reduced (**Supplementary Figure S1**).

Also, there was a significant decrease in the expression of ZO-1 (-75%) and occludin (-50%) 24 h after the TcdA exposure (**Figures 1C,D**), versus their respective vehicle groups. Along with its protective effect on cell viability, rifaximin concentration-dependently increased ZO-1 (0.1, 1.0, and 10 μM rifaximin: 25, 54, and 87%, respectively, vs. TcdA group) and occludin (71, 114, and 262%, respectively, vs. TcdA group) expression. Immunofluorescence analysis (**Figures 1C–E**) showed that rifaximin, at a dose of 10 μM, resulted in an impressive preservation of epithelial barrier architecture, counteracting TcdA-induced decrease in ZO-1 and occludin co-expression in cultured cells (**Figure 1E**). Once again, ketoconazole caused complete loss of the rifaximin-mediated rescue of ZO-1 and occludin proteins (**Figures 1C,D**).

### TLR4/MyD88/NF-κB Expression

**FIGURE 2:**
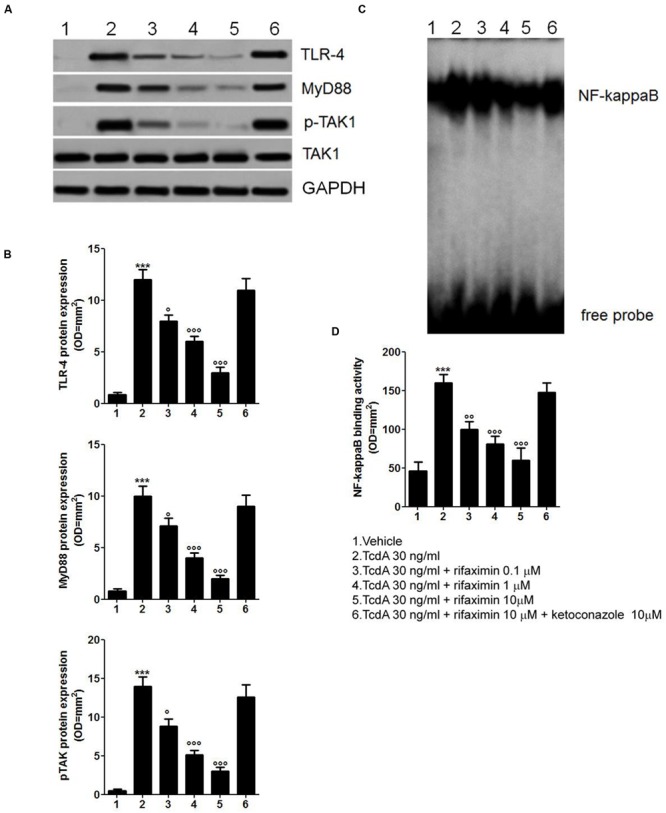
**Rifaximin (0.1, 1.0, and 10 μM) down-regulates the TLR4/MyD88/NF-κB pathway by a PXR-dependent mechanism. (A)** Immunoblot showing the TLR4, MyD88, and phosphorylated/unphosphorylated TAK1 protein bands, and **(B)** Relative densitometric analysis of immunoreactive bands (arbitrary units normalized against the expression of the housekeeping GAPDH protein) showing the effects of rifaximin, given alone or in the presence of ketoconazole (10 μM), on the expression of TLR4, MyD88, and pTAK1 in Caco-2 cell line. **(C)** EMSA analysis showing concentration-dependent inhibition of NF-κB activation by rifaximin, and **(D)** Relative densitometric analysis of NF-κB bands. Results are expressed as mean ± SEM of *n* = 5 experiments performed in triplicate. ^∗∗∗^*p* < 0.001 vs. vehicle group; ^∘∘∘^*p* < 0.001, ^∘∘^*p* < 0.01 and °*p* < 0.05 vs. TcdA group.

There was a significant increase in the expression of TLR4 (1411%) and myeloid differentiation factor 88 (MyD88; 1250%), and phosphorylation of TAK1 (2800%) in the Caco-2 cells 24 h after the TcdA challenge, when compared with the vehicle group (**Figures 2A,B**). The EMSA analysis showed significant up-regulation of NF-κB activity by TcdA versus the vehicle group (348%; **Figures 2A,B**). Rifaximin at 0.1, 1.0, and 10 μM inhibited TLR4 expression (-33, -50, and -75%) and reduced MyD88 (-29, -60, and -81%) and TAK1 expression (-37, -63, and -79%) in a concentration-dependent manner, when compared with the TcdA group (**Figures 2A,B**). Also, rifaximin caused a significant and concentration-dependent decrease in the expression of NF-κB (-38, -50, and -63% at 0.1, 1.0, and 10 μM, respectively, vs. TcdA group; **Figures 2C,D**). These effects of rifaximin were inhibited by ketoconazole (**Figure 2**).

## Discussion

In the present study, treatment with rifaximin significantly increased the TEER in Caco-2 cells in a time-dependent manner when compared with TcdA treatment. Treatment also reduced the cytotoxicity of the TcdA challenge and improved cell viability. Further, rifaximin caused a concentration-dependent decrease in the expression of Bax, caspase-3, and an increase in ZO-1 and occludin expression, and inhibited the expression of TLR4, MyD88, TAK1, and NF-κB in the Caco-2 cells. These effects of rifaximin were inhibited by the PXR antagonist, ketoconazole.

Transepithelial electrical resistance measurement is used as an index of monolayer confluence and integrity in cell culture experiments (Huynh-Delerme et al., 2005). TEER has also been used to measure the paracellular permeability of cell monolayers (Madara et al., 1988). In the present study, TcdA challenge caused a time-dependent marked loss of electrical resistance and barrier integrity of the Caco-2 cells, as seen by the reduction in the TEER after the challenge. Rifaximin treatment improved the TEER values and cell viability in a concentration-dependent manner, demonstrating its efficacy in the prevention of TcdA-induced apoptosis and maintaining barrier integrity. That the effects of rifaximin were inhibited by the PXR antagonist ketoconazole, indicates the mechanism of action of rifaximin involves PXR.

Treatment with rifaximin also caused a decrease in the expression of Bax, and an increase in the expression of ZO-1 and occludin in the Caco-2 cells, in a concentration-dependent manner, and preserved the epithelial barrier architecture in the cultured cells. ZO-1 is a tight junction protein that interacts with the transmembrane protein occludin to maintain the cell barrier integrity (Fanning et al., 1998). while Bax is a protein involved in the promotion of apoptosis (Pawlowski and Kraft, 2000; Westphal et al., 2011) Thus, a decrease in Bax expression should decrease the likelihood of apoptosis, and an increase in ZO-1 and occludin expression should ensure maintenance of barrier integrity, which is what was seen in the present study, with rifaximin effectively maintaining the integrity of the Caco-2 epithelial cell barrier and down-regulating the apoptotic signaling pathway. Again, these effects of rifaximin were completely reversed by ketoconazole, suggesting a PXR-dependent mechanism of action.

Rifaximin treatment also down-regulated the TLR4/MyD88/NF-κB pathway induced by TcdA, through a PXR-dependent mechanism. TLR4 is a transmembrane receptor that is overexpressed in tumor cells (Rakoff-Nahoum and Medzhitov, 2009). TLR4 and its adaptor proteins MyD88 and TAK1 are involved in the activation of the NF-κB pathway causing the release of inflammatory mediators (Akira and Hoshino, 2003; O’Neill et al., 2003; Sato et al., 2005; Kawai and Akira, 2007) In the present study, treatment with rifaximin caused a significant reduction in the expression of TLR4, MyD88, TAK1, and NF-κB after the TcdA challenge, indicating its usefulness in the prevention of TcdA-induced apoptosis by acting on the inflammatory environment. Once again, ketoconazole co-incubation showed complete loss of rifaximin-mediated suppression of these proteins, indicating a role for PXR in these changes.

Pregnane X receptor is a receptor belonging to the nuclear receptor subfamily that are present in the liver and intestine, which are involved in the clearance of xenobiotics from cells (Mani et al., 2013; Smutny et al., 2013). Activation of PXR promotes the expression of several enzymes and transporters that assist in detoxification and removal of xenobiotics, and help in maintaining the integrity of the intestinal barrier (Zhang et al., 2008; Mencarelli et al., 2010). PXR activation also leads to inhibition of the NF-κB pathway and reduces the expression of inflammatory mediators (Cheng et al., 2010; Dou et al., 2012; Zhang et al., 2015) In the present study, reversal of the effects of rifaximin by the PXR antagonist ketoconazole confirm the role of PXR in its mechanism of action. Thus, it appears that rifaximin activates PXR in Caco-2 cells leading to a reduction in TcdA-induced inflammation by down-regulation of the TLR4/MyD88/NF-κB pathway, and improvement of the cell layer integrity.

Rifaximin is a poorly absorbed antibiotic with a favorable safety profile (Scarpignato and Pelosini, 2005). Due to its poor absorption, most of the drug is available in the intestine to locally exert its effects on TcdA-induced apoptosis in the colon. Thus, it may be a promising molecule in the treatment of CDIs.

## Conclusion

Rifaximin effectively inhibited TcdA-induced apoptosis in a cellular model of the intestinal barrier by a PXR-dependent TLR4/MyD88/NF-κB pathway mechanism. Further studies in clinical settings are required to confirm its efficacy in the treatment of CDIs.

## Author Contributions

GS and GE authored the paper and designed the study; NN, SG, LSe, and EC performed the experiments; MP, AdA, and EB performed data analysis and co-authored the paper; RC and LSt contributed to critical revision of the paper. All authors approved the submission of the manuscript.

## Conflict of Interest Statement

The authors declare that the research was conducted in the absence of any commercial or financial relationships that could be construed as a potential conflict of interest. The handling Editor declared a shared affiliation, though no other collaboration, with the authors MP, Ad’A, EB, RC, and GS, and states that the process nevertheless met the standards of a fair and objective review.

## Supplementary Material

FIGURE S1 **(A)** Agarose gel electrophoresis of cultured Caco-2 cell DNA in the presence of TcdA (30 ng/ml) alone or in the presence of increasing concentration of rifaximin (0.1–10 μM) for 24 h. Rifaximin (10 μM) was tested either alone, or in the presence of the PXR antagonist ketoconazole (10 μM). Ketoconazole alone was unable to exert any significant effect on DNA damage. The results are representative of *n* = 3 independent experiments. **(B)** Western blot analysis showing immunoreactive bands referred to the pro-apoptotic active Caspase-3 protein. TcdA (30 ng/ml) induced a significant increase of Caspase-3 expression, that was significantly and concentration-dependently reduced by Rifaximin, whose effect was significantly inhibited by ketoconazole (10 μM). Ketoconazole alone had no pro-apoptotic effect. **(C)** Relative quantification of immunoreactive bands of active caspase-3 protein (arbitrary units). Results are expressed as the mean ± SEM of *n* = 4 experiments performed in triplicate. ^∗∗∗^*P* < 0.001 vs. vehicle group; ^∘∘∘^*P* < 0.001, ^∘∘^*P* < 0.01 vs. TcdA group.

## References

[B1] Akira S. Hoshino K. (2003). Myeloid differentiation factor 88-dependent and -independent pathways in toll-like receptor signaling. *J. Infect. Dis.* 187 S356–S363. 10.1086/374749 12792852

[B2] Cash B. D. Lacy B. E. Rao T. Earnest D. L. (2016). Rifaximin and eluxadoline – newly approved treatments for diarrhea-predominant irritable bowel syndrome: what is their role in clinical practice alongside alosetron? *Expert Opin. Pharmacother.* 17 311–322. 10.1517/14656566.2016.1118052 26559529

[B3] Cheng J. Shah Y. M. Ma X. Pang X. Tanaka T. Kodama T. (2010). Therapeutic role of rifaximin in inflammatory bowel disease: clinical implication of human pregnane X receptor activation. *J. Pharmacol. Exp. Ther.* 335 32–41. 10.1124/jpet.110.170225 20627999 PMC2957776

[B4] Dou W. Mukherjee S. Li H. Venkatesh M. Wang H. Kortagere S. (2012). Alleviation of gut inflammation by Cdx2/Pxr pathway in a mouse model of chemical colitis. *PLoS ONE* 7:e36075 10.1371/journal.pone.0036075 PMC3398007 22815676

[B5] Ebigbo A. Messmann H. (2013). Challenges of *Clostridium difficile* infection. *Med. Klin. Intensivmed. Notfmed.* 108 624–627. 10.1007/s00063-013-0258-7 24129852

[B6] Fanning A. S. Jameson B. J. Jesaitis L. A. Anderson J. M. (1998). The tight junction protein ZO-1 establishes a link between the transmembrane protein occludin and the actin cytoskeleton. *J. Biol. Chem.* 273 29745–29753. 10.1074/jbc.273.45.29745 9792688

[B7] Huynh-Delerme C. Huet H. Noel L. Frigieri A. Kolf-Clauw M. (2005). Increased functional expression of P-glycoprotein in Caco-2 TC7 cells exposed long-term to cadmium. *Toxicol. In Vitro* 19 439–447. 10.1016/j.tiv.2004.08.003 15826803

[B8] Jodlowski T. Z. Oehler R. Kam L. W. Melnychuk I. (2006). Emerging therapies in the treatment of *Clostridium difficile*-associated disease. *Ann. Pharmacother.* 40 2164–2169. 10.1345/aph.1H340 17119105

[B9] Kawai T. Akira S. (2007). Signaling to NF-kappaB by Toll-like receptors. *Trends Mol. Med.* 13 460–469. 10.1016/j.molmed.2007.09.002 18029230

[B10] Layer P. Andresen V. (2010). Review article: rifaximin, a minimally absorbed oral antibacterial, for the treatment of travellers’ diarrhoea. *Aliment. Pharmacol. Ther.* 31 1155–1164. 10.1111/j.1365-2036.2010.04296.x 20331580

[B11] Leﬄer D. A. Lamont J. T. (2015). *Clostridium difficile* infection. *N. Engl. J. Med.* 372 1539–1548. 10.1056/NEJMra1403772 25875259

[B12] Ma X. Shah Y. M. Guo G. L. Wang T. Krausz K. W. Idle J. R. (2007). Rifaximin is a gut-specific human pregnane X receptor activator. *J. Pharmacol. Exp. Ther.* 322 391–398. 10.1124/jpet.107.121913 17442842

[B13] Madara J. L. Stafford J. Barenberg D. Carlson S. (1988). Functional coupling of tight junctions and microfilaments in T84 monolayers. *Am. J. Physiol.* 254 G416–G423. 3279816 10.1152/ajpgi.1988.254.3.G416

[B14] Mani S. Dou W. Redinbo M. R. (2013). PXR antagonists and implication in drug metabolism. *Drug Metab. Rev.* 45 60–72. 10.3109/03602532.2012.746363 23330542 PMC3583015

[B15] Mencarelli A. Migliorati M. Barbanti M. Cipriani S. Palladino G. Distrutti E. (2010). Pregnane-X-receptor mediates the anti-inflammatory activities of rifaximin on detoxification pathways in intestinal epithelial cells. *Biochem. Pharmacol.* 80 1700–1707. 10.1016/j.bcp.2010.08.022 20816942

[B16] Mosmann T. (1983). Rapid colorimetric assay for cellular growth and survival: application to proliferation and cytotoxicity assays. *J. Immunol. Methods* 65 55–63. 10.1016/0022-1759(83)90303-4 6606682

[B17] O’Neill L. A. Dunne A. Edjeback M. Gray P. Jefferies C. Wietek C. (2003). Mal and MyD88: adapter proteins involved in signal transduction by Toll-like receptors. *J. Endotoxin Res.* 9 55–59. 10.1177/09680519030090010701 12691620

[B18] Pawlowski J. Kraft A. S. (2000). Bax-induced apoptotic cell death. *Proc. Natl. Acad. Sci. U.S.A.* 97 529–531. 10.1073/pnas.97.2.529 10639111 PMC33959

[B19] Rakoff-Nahoum S. Medzhitov R. (2009). Toll-like receptors and cancer. *Nat. Rev. Cancer* 9 57–63. 10.1038/nrc2541 19052556

[B20] Rupnik M. Wilcox M. H. Gerding D. N. (2009). *Clostridium difficile* infection: new developments in epidemiology and pathogenesis. *Nat. Rev. Microbiol.* 7 526–536. 10.1038/nrmicro2164 19528959

[B21] Sanchez-Delgado J. Miquel M. (2015). Role of rifaximin in the treatment of hepatic encephalopathy. *Gastroenterol. Hepatol.* 2015 3 [Epub ahead of print]. 10.1016/j.gastrohep.2015.08.003 26545947

[B22] Sato S. Sanjo H. Takeda K. Ninomiya-Tsuji J. Yamamoto M. Kawai T. (2005). Essential function for the kinase TAK1 in innate and adaptive immune responses. *Nat. Immunol.* 6 1087–1095. 10.1038/ni1255 16186825

[B23] Scarpignato C. Pelosini I. (2005). Rifaximin, a poorly absorbed antibiotic: pharmacology and clinical potential. *Chemotherapy* 51 36–66. 10.1159/000081990 15855748

[B24] Scarpignato C. Pelosini I. (2006). Experimental and clinical pharmacology of rifaximin, a gastrointestinal selective antibiotic. *Digestion* 73 13–27. 10.1159/000089776 16498249

[B25] Smutny T. Mani S. Pavek P. (2013). Post-translational and post-transcriptional modifications of pregnane X receptor (PXR) in regulation of the cytochrome P450 superfamily. *Curr. Drug Metab.* 14 1059–1069. 10.2174/1389200214666131211153307 24329114 PMC3914715

[B26] Surawicz C. M. McFarland L. V. (1999). Pseudomembranous colitis: causes and cures. *Digestion* 60 91–100. 10.1159/000007633 10095149

[B27] Terc J. Hansen A. Alston L. Hirota S. A. (2014). Pregnane X receptor agonists enhance intestinal epithelial wound healing and repair of the intestinal barrier following the induction of experimental colitis. *Eur. J. Pharm. Sci.* 55 12–19. 10.1016/j.ejps.2014.01.007 24486481

[B28] Waltz P. Zuckerbraun B. (2016). Novel therapies for severe *Clostridium difficile* colitis. *Curr. Opin. Crit. Care* 10.1097/MCC.0000000000000282 [Epub ahead of print]. 26771898

[B29] Wan Y. C. Li T. Han Y. D. Zhang H. Y. Lin H. Zhang B. (2015). Effect of pregnane xenobiotic receptor activation on inflammatory bowel disease treated with rifaximin. *J. Biol. Regul. Homeost. Agents* 29 401–410. 26122229

[B30] Wells C. L. van de Westerlo E. M. Jechorek R. P. Haines H. M. Erlandsen S. L. (1998). Cytochalasin-induced actin disruption of polarized enterocytes can augment internalization of bacteria. *Infect. Immun.* 66 2410–2419. 9596696 10.1128/iai.66.6.2410-2419.1998 PMC108218

[B31] Westphal D. Dewson G. Czabotar P. E. Kluck R. M. (2011). Molecular biology of Bax and Bak activation and action. *Biochim. Biophys. Acta* 1813 521–531. 10.1016/j.bbamcr.2010.12.019 21195116

[B32] Zhang B. Xie W. Krasowski M. D. (2008). PXR: a xenobiotic receptor of diverse function implicated in pharmacogenetics. *Pharmacogenomics* 9 1695–1709. 10.2217/14622416.9.11.1695 19018724 PMC2593625

[B33] Zhang J. Ding L. Wang B. Ren G. Sun A. Deng C. (2015). Notoginsenoside R1 attenuates experimental inflammatory bowel disease via pregnane X receptor activation. *J. Pharmacol. Exp. Ther.* 352 315–324. 10.1124/jpet.114.218750 25472953 PMC4293438

