# Supplementary figures and images for: Rifaximin Improves Clostridium difficile Toxin A-Induced Toxicity in Caco-2 Cells by the PXR-Dependent TLR4/MyD88/NF-κB Pathway
Source: Front Pharmacol. 2016 May 9;7:120. doi: 10.3389/fphar.2016.00120 (PMC4860461; doi:10.3389/fphar.2016.00120)

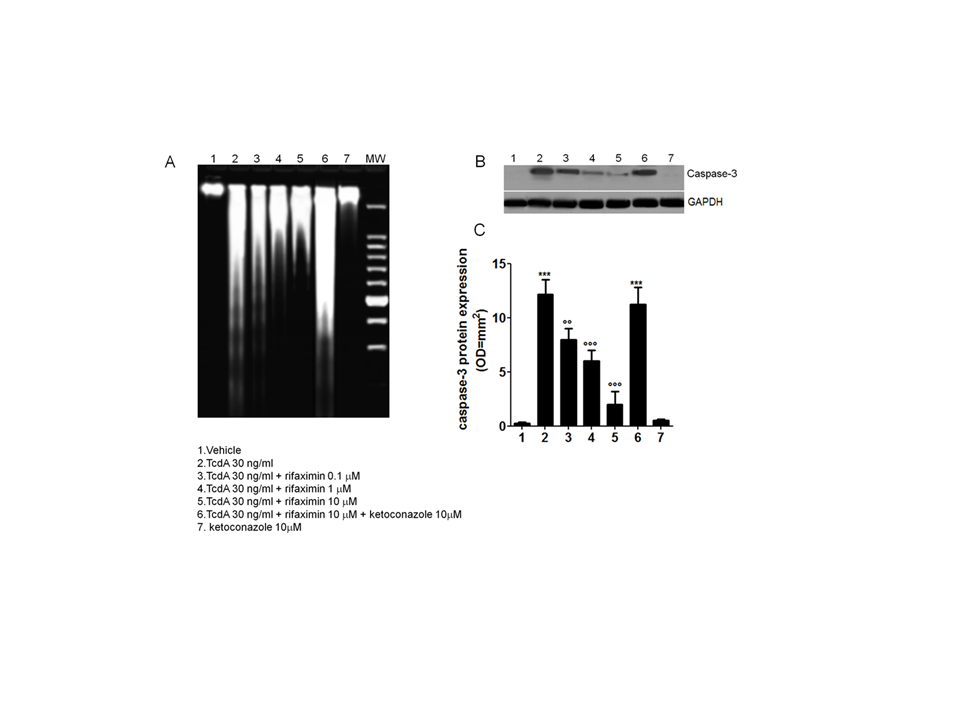

Supplement: FIGURE S1 — (A) Agarose gel electrophoresis of cultured Caco-2 cell DNA in the presence of TcdA (30 ng/ml) alone or in the presence of increasing concentration of rifaximin (0.1–10 μM) for 24 h. Rifaximin (10 μM) was tested either alone, or in the presence of the PXR antagonist ketoconazole (10 μM). Ketoconazole alone was unable to exert any significant effect on DNA damage. The results are representative of n = 3 independent experiments. (B) Western blot analysis showing immunoreactive bands referred to the pro-apoptotic active Caspase-3 protein. TcdA (30 ng/ml) induced a significant increase of Caspase-3 expression, that was significantly and concentration-dependently reduced by Rifaximin, whose effect was significantly inhibited by ketoconazole (10 μM). Ketoconazole alone had no pro-apoptotic effect. (C) Relative quantification of immunoreactive bands of active caspase-3 protein (arbitrary units). Results are expressed as the mean ± SEM of n = 4 experiments performed in triplicate. ∗∗∗P < 0.001 vs. vehicle group; ∘∘∘P < 0.001, ∘∘P < 0.01 vs. TcdA group. [file Image_1.TIF]
